# Levothyroxine Treatment in Pregnancy: Indications, Efficacy, and Therapeutic Regimen

**DOI:** 10.4061/2011/843591

**Published:** 2011-08-25

**Authors:** Joanna Klubo-Gwiezdzinska, Kenneth D. Burman, Douglas Van Nostrand, Leonard Wartofsky

**Affiliations:** ^1^Section of Endocrinology, Department of Medicine, Washington Hospital Center, Washington, DC 20010, USA; ^2^Division of Endocrinology and Medicine, Georgetown's University Hospital, Washington, DC 20007, USA; ^3^Nuclear Medicine Division, Washington Hospital Center, Washington, DC 20010, USA

## Abstract

The prevalence of overt and subclinical hypothyroidism during pregnancy is estimated to be 0.3–0.5% and 2–3%, respectively. Thyroid autoantibodies are found in 5–18% of women in the childbearing age. The aim of this review is to underscore the clinical significance of these findings on the health of both the mother and her offspring. Methods of evaluation of thyroid function tests (TFTs) during pregnancy are described as are the threshold values for the diagnosis of overt and subclinical hypothyroidism or hypothyroxinemia. Anticipated differences in TFTs in iodine-sufficient and iodine-deficient areas are discussed and data are provided on potential complications of hypothyroidism/hypothyroxinemia and autoimmune thyroid disease during pregnancy and adverse effects for the offspring. The beneficial effects of levothyroxine therapy on pregnancy outcomes and offspring development are discussed with a proposed treatment regimen and follow up strategy.

## 1. Introduction

During normal gestation, thyroid hormone production is augmented in order to meet the increased physiologic demands of the growing fetal placental unit. Alterations in thyroid function with pregnancy are derived via several mechanisms. Notably, there is an increase in serum estrogen levels during the first half of gestation up to 500–1000 pg/mL, resulting in upregulation by two- or threefold of hepatic production of thyroxine binding globulin (TBG) [1, 2]. The increased TBG levels alter the equilibrium between bound and free thyroxine (FT4) causing a temporary reduction in FT4 that in turn leads to increased thyrotropin (TSH) stimulation of the thyroid gland and physiologic restoration of FT4 at the cost of higher serum total T4 (TT4) levels. A second factor is the increased placental production of human chorionic gonadotropin (hCG), reaching a peak of approximately 50,000–75,000 IU/L at 8–11 weeks. This is significant because of the direct stimulatory effect of hCG on thyrocytes that is mediated through binding to the TSH receptor. Yet a third issue is related to the increased need for iodine in pregnancy that is required to fuel the increases in thyroid hormone synthesis and compounded by the loss of iodine due to enhanced renal clearance [3, 4]. Therefore, the recommended average iodine intake during the pregnancy is between 250 and 500 ug/d [5, 6]. A final factor is the presence of placental iodothyronine deiodinase type III which alters the metabolism, distribution, and availability of T4 for both mother and fetus [4].

The prevalence of hypothyroidism during pregnancy is estimated to be 0.3–0.5% for overt hypothyroidism (OH) and 2-3% for subclinical hypothyroidism (SH). Thyroid autoantibodies are found in 5–18% of women in the childbearing age, and chronic autoimmune thyroiditis (AITD) is the main cause of hypothyroidism during pregnancy in iodide sufficient areas [7–9]. However, on a worldwide basis the most important cause of thyroid insufficiency remains iodine deficiency [10].

In view of the frequency of either OH or SH during pregnancy and the associated altered physiology, several questions face clinicians managing subjects with suspected thyroid dysfunction during pregnancy including (1) what are the threshold values for the diagnosis of overt hypothyroidism (OH), subclinical hypothyroidism (SH), or hypothyroxinemia during the pregnancy? (2) Is the interpretation of thyroid function tests different in iodine-sufficient than in iodine-deficient areas? (3) What are the complications of hypothyroidism/hypothyroxinemia and autoimmune thyroid disease during the pregnancy? (4) Is levothyroxine therapy beneficial and effective in regard to improved outcomes and reduced complications associated with pregnancy and delivery? (5) How to select the patients for whom the treatment may be beneficial? (6) What is the appropriate treatment regimen and what are target thyroid function test values and how often should they be monitored? The aim of this paper is to address all of the above mentioned questions.

## 2. Methods

We have searched PubMed database from January 1970 to January 2011 for the articles written in English using the following keywords: “pregnancy and thyroid function”, “pregnancy and hypothyroidism”, “pregnancy and subclinical hypothyroidism”, “pregnancy and hypothyroxinemia”, “pregnancy and levothyroxine treatment”, “pregnancy and iodine deficiency”, “offspring complications and hypothyroidism”, “offspring complications and hypothyroxinemia” and “offspring complications and iodine deficiency”. We have included retrospective and prospective observational studies, clinical trials, meta-analyses, review papers, and guidelines published in the indexed journals.

### 2.1. What are the Threshold Values for the Diagnosis of Overt Hypothyroidism (OH), Subclinical Hypothyroidism (SH), or Hypothyroxinemia during the Pregnancy? 

Establishment of reference ranges for thyroid function tests during pregnancy has been problematic due to variables based upon age, smoking status, ethnicity, BMI, iodine nutritional status, and the presence of latent or overt autoimmune thyroid disease [8, 11]. 

Interpretation of any given value for FT4 or TSH should take into account the possible differences between population based reference ranges of thyroid function tests and a given patient's narrower individual reference range. Based on data from 877 pregnant women, Shields et al. suggested that an individual's level of TSH is associated with variation in the PDE8B gene with AA genotypic women being more likely to have elevated TSH concentrations (>4.21 mIU/L) compared to women with AG or GG genotypes (9.6 versus 3.5%, *P* < 0.0004). This observation was independent of the presence or absence of autoimmune thyroid disease (AITD) [12].

#### 2.1.1. Normal Absolute Values and Optimal Methods for Assessment of Thyroid Function during Pregnancy

**TSH**

TSH is the single best indicator of insufficient thyroid hormone due to primary hypothyroidism [6]. However, there is a necessity for the trimester-specific reference range for TSH in each laboratory or at least each country/region in order to properly interpret the thyroid function tests.

Guidelines for diagnosis developed by the Endocrine Society and endorsed by the American Thyroid Association in 2007 recommend that TSH values should be <2.5 mIU/L in the first trimester and <3 mIU/L in the second and third trimester [6]. Data supporting this recommendation were derived from observations of the consistency in TSH ranges for first-trimester thyroperoxidase antibody-negative women, with a consensus centering around a lower limit of normal of 0.04 and upper range of normal of 2.5 mIU/L. It is worthwhile to underscore that this reference range was not significantly different between various populations. In a prospective study of 343 Chinese women, Panesar et al. [13] noted a normative range for first-trimester TSH levels of 0.03–2.3 mIU/L, which did not differ significantly from the range of 0.02–2.15 mIU/L established by Gilbert et al. [14] in 1817 Australian women between 9 and 13 weeks of gestation. A somewhat wider range and higher upper limit was seen by Pearce et al. [15] of 0.04–3.6 mIU/L in 585 thyroid antibody-negative women before 14 wks gestation, and by Stricker et al. [16] after screening 783 thyroid antibody-negative women in Switzerland (TSH 0.08–2.83 mIU/L). Männistö et al. based on a large (*n* = 9362) prospective population-based cohort from Northern Finland without AITD and with sufficient iodine intake reported a TSH reference range of 0.07 to 3.1 in the first trimester and up to 3.5 mIU/L in the second trimester. Moreover, they also observed that thyroid hormone levels are affected by BMI with higher TSH and FT3 and lower FT4 concentrations observed in obese women [11].

**FT4, FT3**

Although equilibrium dialysis and mass spectrometry/gas chromatography are the gold standards and are the most reliable methods of measurement of both FT4 and FT3 concentrations, these methods are too technically complex and expensive for routine use. Consequently, most FT4 testing in clinical laboratories is based on two-step or labeled antibody methodology, which is sensitive to abnormal TBG levels and liable to error [17, 18]. There is a need for a method-specific, trimester-specific, and possibly population-specific FT4 reference range. Männistö et al. addressed this question for the anti-TPO negative and iodine sufficient Caucasian population, documenting that FT4 measured with chemiluminescent immunoassay increases slightly during early pregnancy and then decreases with the reference ranging being between 11 and 22 pmol/L. Reference intervals for FT3 in the same study were stable during the pregnancy and ranged from 3.4 to 7 pmol/L [11]. However, Lee et al. documented that FT4 measured by two different immunometric assays diverges so significantly during the second and third trimesters that the vast majority of women would be diagnosed incorrectly as hypothyroxinemic by laboratory criteria alone. Each specific immunoassay needs to have normals and abnormals determined for the pregnant state, or immunoassays may underestimate FT4 [19].

**TT4**

The TT4 increase in pregnancy is more predictable than alterations in FT4, being generally 1.5 times nonpregnant levels which is primarily related to increases in serum TBG as described above. Many studies have shown remarkably consistent ranges for T4 throughout pregnancy—approximately 143–158% of nonpregnant values. Adjusting the TT4 in pregnancy by a factor of 1.5 compared with nonpregnant reference ranges is a good reflection of FT4 [19]. Therefore, some authors advocate the use of TT4 in preference to FT4 for the evaluation and management of pregnant patients [20]. The Endocrine Society Guidelines as well as Laboratory Medicine Practice Guidelines advocate a TT4 cutoff of 100 nmol/L as appropriate for detecting a low FT4 state in pregnancy [6, 20].

**FT4 Index**

The free thyroxine index (FT4I) is measured as total T4 mathematically corrected for thyroxine binding globulin (TBG). FT4I is calculated by dividing TT4 by the thyroid hormone-binding ratio—the estimate of TBG. Changes in FT4I are consistent with the expected effects of TBG and hCG during pregnancy with a physiologic increase in the first trimester with normalization to nonpregnant levels in the second and third trimesters. This pattern of change during pregnancy corresponds to that described using the gold standard FT4 methods of equilibrium dialysis and tandem mass spectrometry [19].

To summarize, the FT4I index or the TT4 adjusted for pregnancy are reliable methods of estimating free thyroxine status in pregnancy.

### 2.2. Is the Interpretation of Thyroid Function Tests Different in Iodine-Sufficient than in Iodine-Deficient Areas? 

Approximately 1.9 billion individuals, including 285 million school-aged children, are estimated to have inadequate iodine nutrition. Severe iodine deficiency in pregnancy can cause hypothyroidism, poor pregnancy outcome, cretinism, and irreversible mental retardation. Mild-to-moderate iodine deficiency *in utero *and in childhood may result in less severe learning disability, poor growth, and diffuse goiter [21]. It has been also suggested that even mild iodine deficiency may be associated with attention deficit and hyperactivity disorders in offspring [22]. 

The prevalence of iodine deficiency is lowest in the Americas (10.1%) and highest in Europe (59.9%) [23]. The thyroid gland responds to iodine deficiency through regulatory mechanisms that include decreased synthesis and secretion of T4 in favor of T3. In the case of mild to moderate iodine deficiency during the pregnancy, the circulating T3 levels remain normal or even increase slightly and circulating TSH levels do not increase. So the thyroid function tests may misleadingly indicate euthyroidism, while the amount of T4 available for the fetus might be insufficient [24, 25]. Another commonly seen diagnostic marker of iodine deficiency is an elevated serum thyroglobulin level [26]. Serum Tg is well correlated with the severity of iodine deficiency as measured by UI [27]. Intervention studies examining the potential of Tg as an indicator of response to iodine supplementation revealed that Tg falls rapidly with iodine repletion and that Tg is a more sensitive indicator of iodine repletion than TSH or T4 [28, 29].

Iodine repletion in severely iodine-deficient pregnant women or infants may reduce the infant mortality rate by at least 50% [21]. A blinded, placebo-controlled clinical trial conducted in the 1960s in Papua, New Guinea, demonstrated that preconception supplementation of severely iodine-deficient women with iodinated oil eliminated the risk for cretinism and improved offspring cognitive function and survival [30]. These findings have subsequently been replicated in many regions of the world, indicating that iodine supplementation in severely iodine-deficient regions may increase the average child IQ by 12.5 points [31, 32]. 

Iodine supplementation of moderately deficient pregnant women appears to consistently decrease maternal and neonatal thyroid volumes and Tg levels [31]. Effects on maternal thyroid function have been variable, with significant maternal TSH decreases seen only in four of eight published studies and with increases in maternal T4 or FT4 noted in two studies [31]. The observed differences in the response to iodine supplementation may be related to the onset of intervention. In a prospective observational study, Moleti et al. studied 433 euthyroid anti-TPO antibody-negative women and observed TSH increases during gestation in 26.1% women taking 150 ug iodine supplementation during the pregnancy compared to 15.6% of women with a history of iodinated salt intake several months before the pregnancy (*P* < 0.05). However both methods of iodine supplementation were sufficient to reduce the proportion of pregnant women with hypothyroxinemia, which was observed in 8.3% of women taking 150 ug iodine supplements, 9.5% of women with a history of iodine salt use, and 20% of women without any iodine supplementation [33]. These observations were confirmed in other studies [34, 35]. Neurodevelopmental outcomes were improved in children whose mothers received iodine supplementation early in pregnancy and were lost if supplementation was started after the 10th week of pregnancy [36, 37]. 

These results suggest that women from mildly and moderately iodine-deficient areas, which include several European countries, should be advised to start iodine supplementation several months prior to conception in order to saturate intrathyroidal iodine stores. Ideally, women should have adequate intrathyroidal iodine stores (10–20 mg) before conception. Unfortunately, well-conducted randomized maternal iodine-supplementation studies with long-term follow-up data on psychomotor and mental development of children are lacking.

### 2.3. What are the Complications ofHypothyroidism/Hypothyroxinemia and Autoimmune Thyroid Disease during the Pregnancy? 

#### 2.3.1. Hypothyroidism

**Pregnancy Complications**

There is a known association between hypothyroidism and decreased fertility, as well as increased risk for early and late obstetrical complications, such as increased prevalence of abortion, anemia, gestational hypertension, placental abruption, and postpartum hemorrhages. As would be expected, these complications are more frequent with OH than with SH [38–43]. In one study of 216 women with early miscarriage, SH and AITD were independently associated with miscarriage with SH being specifically associated with very early embryo loss at 6.5 weeks [44]. In contrast, a prospective study of 10,990 women in US and Ireland with biochemical evidence of SH did not reveal excessive adverse pregnancy outcomes [45]. Recent studies from The Netherlands documented an association between TSH levels exceeding >2.4 mIU/mL between the 35th and 38th week of gestation are associated with the approximately 2-fold increased risk for breech presentation [46, 47]. Elevated TSH levels earlier during the pregnancy were not associated with risk of breech presentation. Interestingly, higher levels of TSH at end term were independently associated with the lack of successful outcome of external cephalic version [48].

**Adverse Outcomes for the Neonate/Offspring**

Untreated maternal OH is associated with adverse neonatal outcomes including premature birth, low birth weight, and neonatal respiratory distress. Although less frequent than with OH, complications have also been described in newborns from mothers with SH, including a doubling of the rate of preterm delivery [49] in pregnant women before 20 wk gestation. Stagnaro-Green et al. [50] compared the thyroid status of women with preterm delivery to matched controls who delivered at term and found a 3-fold increase in the incidence of SH in the women with very early preterm deliveries (before 32 wks).

Four decades ago Man et al. [51, 52] observed that children born to mothers with inadequately treated hypothyroidism had significantly reduced intelligence quotients (IQs). The first large-scale prospective study on the outcome of children born to mothers with SH during pregnancy was reported by Haddow et al. in 1999 [53]. In this study, extensive neuropsychological testing of the school-age children revealed that children born to women with untreated SH had on average an IQ score that was 7 points below the mean IQ of children born to healthy women and thyroxine-treated women. Furthermore, there were three times as many children with IQs that were 2 SD scores below the mean IQ of controls in the children born to untreated women with SH.

Of note, there is a specific type of combined maternal and fetal hypothyroidism during gestation associated with the presence of TSH receptor blocking antibodies in women with AITD. This entity is associated with more severe cognitive delay in the offspring than seen with either fetal or maternal hypothyroidism alone, probably because maternal T4 is unavailable to compensate for the fetal hypothyroidism. This disorder should be suspected if unusually high doses of levothyroxine (LT4) are required to normalize maternal thyroid function during gestation [54]. Additional studies will be required to elucidate the effect of SH on the long-term neuropsychological development of offspring.

#### 2.3.2. Isolated Hypothyroxinemia

**Pregnancy Complications**

Cleary-Goldman et al. showed that isolated hypothyroxinemia, which was observed in 232 of 10,990 pregnant women, is not associated with adverse pregnancy outcomes [45]. Similar observations were noted by Casey et al. who observed hypothyroxinemia among 233 of 17,298 pregnant women. This was not associated with any increased risk for adverse pregnancy outcomes in this subpopulation [55].

**Adverse Outcomes in Offspring**

Despite the limitations of FT4 assays, multiple studies have demonstrated that low-normal FT4 concentrations are associated with adverse outcomes in the offspring. Pop et al. investigated the developmental outcome in children born to women with isolated low T4 levels in the first trimester of pregnancy, defined as values within the lowest 10th decile of “normal” pregnant T4 values [56]. Results suggested that isolated hypothyroxinemia is associated with a lower developmental index in the children at approximately 10 months of age. This observation was confirmed later by the same group based on a larger cohort and more refined motor and mental evaluations in infants aged 1 and 2 yrs [57]. They documented that children born to mothers with prolonged levels of low T4 (until wk 24 or later) showed an 8- to 10-point deficit for motor and mental development compared to infants of women whose serum FT4 levels recovered spontaneously to normal later in gestation.

These results were confirmed by Vermiglio et al. [22], who compared the neuropsychological development of children of mothers from a moderately iodine-deficient area to that of children of mothers from a marginally iodine-sufficient area. The offspring of the mothers with lower FT4 values during gestation were found to have an increased incidence of attention deficit and hyperactivity disorder as well as a reduced IQ, compared with controls. Similarly, Kooistra et al. observed that newborns from hypothyroxinemic mothers (FT4 below the 10th percentile at 12th week of pregnancy), and evaluated 3 weeks after delivery with the Neonatal Behavioural Assessment Scale, had significantly lower orientation index scores compared to children whose mothers had FT4 levels between the 50th and 90th percentiles [58]. Similar observation was found in Chinese population, indicating that children of women with either SH, hypothyroxinemia, or elevated TPO Ab titres at 16–20 weeks gestation had mean intelligence and motor scores significantly lower than controls [59]. Finally, a recent population-based cohort study from The Netherlands involving 3659 children and their mothers documented that both mild (below the 10th percentile) and severe (below 5 percentile) maternal hypothyroxinemias were associated with a higher risk of expressive language delay at 18 and 30 months. Severe maternal hypothyroxinemia also predicted a higher risk of nonverbal cognitive delay [60].

#### 2.3.3. Euthyroid Autoimmune Thyroid Disease

**Pregnancy Complications**

Experimental studies on pregnant mice have shown an increased rate of miscarriage after immunization with Tg [61, 62]. Several studies have also indicated an association in women between the presence of AITD and an increased miscarriage rate and preterm delivery [63–65]. Three hypotheses have been proposed to explain this association: (1) thyroid antibodies may represent a marker of a generalized autoimmune imbalance that increases risk of miscarriage; (2) preexisting subtle thyroid dysfunction due to AITD may worsen during pregnancy; (3) because women with AITD have a higher prevalence of infertility, they may be older than those without AITD and thus be more prone to fetal loss. Women with AITD are also more likely to suffer from postpartum thyroiditis and postpartum depression [66–69].

**Side Effects in the Offspring**

As far as we could determine, other than the one study cited above [59], there is no other evidence that high maternal TPO Ab titers are associated with a delayed neurologic development of the offspring.

#### 2.3.4. Euthyroid TPO Ab Negative Women with TSH between 2.5 and 5 mIU/L

**Pregnancy Complications**

A higher rate of spontaneous pregnancy loss was observed in a study of 642 women with serum TSH ranging between 2.5 and 5 mIU/L in the first trimester than in 3481 women with TSH below 2.5 (6.1% versus 3.6%, *P* = 0.006, resp.) [70].

### 2.4. Is Levothyroxine Therapy Beneficial and Effective in regard to Improved Outcomes and Reduced Complications Associated with Pregnancy and Delivery? 

Based upon data in the study by Abalovich et al. [38] focused on 150 pregnancy outcomes in 114 women who had either OH (*n* = 52) or SH (*n* = 62) (Table 1), Stagnaro-Green analyzed the preterm delivery rate of women who were either adequately treated at conception (*n* = 99) or during pregnancy (*n* = 27) versus women who were inadequately treated during pregnancy (*n* = 24). The analysis revealed a significantly lower preterm delivery rate of 1.6% after adequate treatment with L-T4 compared to a rate of 12.5% in the group of inadequately treated women with TSH > 4 mIU/L during pregnancy (*P* < 0.05) [74]. There is also evidence that LT4 treatment can improve implantation rate and live birth rate in infertile women with subclinical hypothyroidism undergoing in vitro fertilization [75].

Rovet [76] investigated children up to the age of 5 born to women who, although having been treated for hypothyroidism during pregnancy, received suboptimal LT4 dosage as indicated by mean TSH levels between 5 and 7 mIU/liter. The children at preschool age were found to have a mild reduction in global intelligence that was inversely correlated with maternal TSH level during the third trimester. No negative impact was noted on language, visual spatial ability, fine motor performance, or preschool ability.

Preliminary results of the “Controlled Antenatal Thyroid Screening Study” (CATS) were presented in September 2010 during International Thyroid Congress [73]. The CATS trial was a prospective randomized study that screened 22,000 women within the 16th week of gestation for thyroid status with TSH and FT4 measurements. Women with FT4 values lower than the 2.5th percentile and/or TSH values above the 97.5th percentile were randomly assigned into an intervention group treated with LT4 or a control group without intervention. Neuropsychological development assessed by Wechsler Preschool and Primary Scale of Intelligence (WPPS III) was performed in the offspring of both groups at 3 years of age. Primary outcomes consisted of the mean WPPS III score and the percentage of offspring with IQ of <85 points. A primary analysis that included an intention to treat analysis revealed no significant differences. The secondary analysis which excluded women who had been noncompliant with LT4 treatment also revealed no difference in relative risk of full-scale IQ being below 85 in the screened group compared to the control group.

A second study is presently in progress under the auspices of the NIH. Pregnancy screening was initiated in October 2006, and study completion is anticipated in 2015. That study will eventually comprise a total of 120,000 pregnant women, recruited from an obstetrical US network of 14 institutions. Women with SH or isolated hypothyroxinemia will be randomized to placebo versus LT4 treatment to normalize serum TSH in women with SH or to normalize serum FT4 in women with isolated hypothyroxinemia [77]. The primary outcome of the study will be intellectual function of children at 5 years of age as measured by the WPPSI-III. The WPPSI-III scores of progeny of treated women will be compared to the children of untreated women. Secondary outcomes of the study include assessment of fetal growth, rates of preterm delivery, preeclampsia, placental abruption, stillbirth, and development of postpartum thyroid dysfunction.

#### 2.4.1. Euthyroid Women with AITD

Negro et al. published the first study focused on the possible benefit of LT4 treatment of anti-TPO positive euthyroid women defined as having serum TSH within a range 0.27–4.2 mIU/L [72]. Among 984 patients who completed the study, there were 155 anti-TPO positive women randomized into an intervention group (*n* = 57) treated with LT4 at their first prenatal visit performed at a median 10 weeks of gestation and a no intervention group (*n* = 58). TPO Ab negative women (*n* = 869) served as a normal control group. This study importantly demonstrated salutary effects of LT4 administration to both correct maternal thyroid dysfunction and also decrease the rate of adverse obstetrical events such as miscarriage and premature delivery, bringing their prevalence down to those of the control population (Table 1). No study has yet demonstrated whether similar benefit might be gained with LT4 therapy of TPO Ab negative women.

### 2.5. How to Select the Patients for Whom the Treatment May Be Beneficial?

#### 2.5.1. Screening for Thyroid Dysfunction during Pregnancy

In view of the growing evidence that abnormal thyroid function during pregnancy is associated with less optimal outcomes that can be improved with LT4 treatment, the question arises as to whether to screen in early pregnancy for thyroid dysfunction. The 2007 Endocrine Society Guidelines [6] recommend case finding by measurement of TSH in women in any of the following categories:

1. personal history of abnormal thyroid function,
2. family history of thyroid disease,
3. goiter,
4. positive thyroid antibodies,
5. symptoms or clinical signs suggestive of thyroid dysfunction,
6. type 1 diabetes,
7. other autoimmune disorders,
8. infertility,
9. history of therapeutic head or neck irradiation,
10. history of miscarriage or preterm delivery.

However, it has been suggested that we might fail to detect 30% of hypothyroid and 69% of hyperthyroid women if only high-risk pregnant women are screened [78]. This argument is supported by demonstrating that screening a low-risk group identified both hypothyroidism and hyperthyroidism and allowed early therapy that resulted in a lower rate of adverse obstetrical and fetal outcomes [79]. While a major argument against screening is the associated cost of TSH measurements, it has been demonstrated that screening pregnant women with TSH in the first trimester of pregnancy is cost-saving compared with no screening and screening by measurement of anti-TPO antibodies is also an economically favorable screening strategy [80].

### 2.6. What is the Appropriate Treatment Regimen; What are Target Thyroid Function Test Values, and How Often Should They Be Monitored? 

Once a diagnosis of either OH or SH is made during pregnancy, the Guidelines of the Endocrine Society clearly recommend initiation of treatment with LT4 [6].

*“LT4 dose often needs to be incremented by 4–6 wk gestation and may require a 30–50% increment in dosage. If overt hypothyroidism is diagnosed during pregnancy, thyroid function tests should be normalized as rapidly as possible. The target threshold TSH should be less than 2.5 mIU/L in the first trimester and less than 3 mIU/L in second and third trimesters or to trimester-specific normal TSH ranges. Thyroid function tests should be remeasured within 30–40 d. After delivery, most hypothyroid women need to decrease the thyroxine dosage they received during pregnancy.”* *[6]*

Notwithstanding the data extant demonstrating efficacy of LT4 therapy in preventing adverse pregnancy and offspring outcomes and the availability of published guidelines addressing the treatment strategy, there is evidence that 24–49% of women treated with LT4 before conception still have elevated TSH levels at their first prenatal visit [71, 81, 82]. The reasons for this are not clear but could include failure to appreciate and recognize that dosage requirements for LT4 may change with pregnancy as well as perhaps failure to remeasure TSH at sufficiently frequent intervals. In women already taking LT4, the magnitude of increase in LT4 requirements with pregnancy is approximately 40–50% of the prepregnancy dosage in athyreotic patients and about 20–30% for patients with underlying Hashimoto's thyroiditis [6, 83–89]. The difference is due to the fact that the latter patients typically will have some residual functioning mass of thyroid tissue capable of releasing T4 that complements the daily exogenous LT4 dose. 

Because of the potential for clinicians to fail to identify the demand for an increased LT4 dosage in pregnancy, several studies have aimed to identify a practical therapeutic approach to address this issue [90]. Rotondi et al. [91] pointed out that the intervention should be made preconception. They prospectively examined 25 women with compensated hypothyroidism of different etiology anticipating pregnancy and assigned them to two groups: 14 patients had their LT4 dose increased to a partially suppressive dose, while 11 patients continued the same therapeutic regimen. Their results indicated that a preconception dosage of LT4 targeted at TSH in the lower quartile of the reference range may result in adequate maternal thyroid function up to the first postconception evaluation. This observation is in agreement with a retrospective study by Abalovich et al. of 53 women with compensated hypothyroidism defined as a TSH <2.5 mIU/L six months prior to conception. When the preconception TSH was below 1.2 mIU/mL, only 17.2% of women required incremental LT4 adjustment during the pregnancy compared to 50% of women having a preconception TSH between 1.2 and 2.4 mIU/mL (*P* < 0.02) [92].

These data support the logic of the premise that women will need varying degrees of adjustment of their prepregnancy LT4 dosage based upon the underlying cause of their thyroid dysfunction. The best example of this was demonstrated by Loh et al. [93] who observed that patients with a history of thyroid cancer on doses of LT4 sufficient to suppress preconception TSH required smaller and less frequent incremental adjustments of LT4 during the pregnancy in order to keep TSH within the normal range than did patients suffering from other causes of hypothyroidism. Some investigators have proposed a simple and practical formula to address this issue, suggesting that hypothyroid women on LT4 should be advised that once pregnant they should increase their LT4 dose by about 25% by taking two extra doses per week of their usual daily dose of LT4 [94]. Other than the consideration of the cost of additional TSH measurements throughout pregnancy, we would propose that assurance of euthyroidism during pregnancy is best obtained by an individualized approach to LT4 dosage adjustment based on a TSH measurement done every 2 weeks during the first trimester and then less frequently thereafter as suggested by Burman [95].

The very recent THERAPY trial (Thyroid Hormone Early Adjustment in Pregnancy) proposed another approach [96]. Sixty women with treated hypothyroidism were prospectively randomized before their anticipated pregnancy to one of two groups who received an increased LT4 dose of either 2 or 3 tablets per week once pregnancy was confirmed. Enrollment took place at a median 5.5 weeks of pregnancy, and patients were followed with the measurements of TSH, TT4, and thyroid hormone binding ratio every 2 weeks through week 20 and then once again at 30 weeks. Interestingly, despite the early enrollment, nearly 30% of women were already hypothyroid. The authors documented that increasing LT4 by 2 tablets per week resulted in the achievement of TSH below 2.5 mIU/L during the first trimester in 85% of patients without a significantly increased risk of iatrogenic hyperthyroidism, which was observed in 2/25 patients compared with the group receiving a 3-tablet per week increment, in which suppressed TSH was observed in 14/23 women. This study also assessed the optimal follow-up strategy throughout the remainder of pregnancy; documenting that 92% of abnormal TSH values would be detected by testing every 4 weeks. 

 Based upon this review of the literature, it appears clear that there is a necessity to both update and promulgate novel guidelines in regard to LT4 treatment during pregnancy. In addition to the endocrine community, the guidelines importantly should reach a target population of obstetricians and family care physicians who provide the majority of antenatal care. Indeed, according to one study of obstetricians and general practitioners in Wisconsin, there is currently a limited awareness of the 2007 Endocrine Society Guidelines in only 11.5% of the latter population of caregivers [97].

#### 2.6.1. A Future Research Agenda That Could Illuminate Remaining Aspects of the Care of Pregnant Women with Thyroid Dysfunction Might Include the Following

1. Determination of which strategy is most appropriate, universal screening, or case finding, based on large prospective trials like CATS and NIH trial.
2. Assessment of the best screening strategy (TSH versus anti-TPO Ab versus TSH + anti-TPO Ab) in different populations characterized by various iodine nutritional status.
3. Examining the effects of LT4 treatment of SH and isolated hypothyroxinemia on the long-term intellectual development of offspring.
4. A study of the efficacy of LT4 treatment of TPO Ab negative women with preconception TSH levels >2.5 mIU/L.
5. Confirmation of whether the optimal preconception target TSH concentration is 1.2 mIU/L versus 1.2–2.5 mIU/L.

## 3. Addendum

After the acceptance of this review for the publication, the new Guidelines of the American Thyroid Association for the Diagnosis and Management of Thyroid Disease During Pregnancy and Postpartum were released online [98]. 

The content of this review is concordant with ATA guidelines in the following recommendations.

1. Trimester and population specific reference ranges for TSH should be applied. If they are not available in the laboratory, the following reference ranges are recommended: first trimester, 0.1–2.5 mIU/L; second trimester, 0.2–3.0 mIU/L; third trimester, 0.3–3.0 mIU/L.
2. Method-specific and trimester-specific reference ranges of serum FT4 are required.
3. All women with hypothyroidism and women with subclinical hypothyroidism who are positive for TPOAb should be treated with LT4; however due to the lack of randomized controlled trials there is insufficient evidence to recommend for or against universal LT4 treatment in TPO Ab negative pregnant women with subclinical hypothyroidism.
4. The goal of LT4 treatment is to normalize maternal serum TSH values within the trimester-specific pregnancy reference range.
5. LT4 dose should be increased by 25–30% upon a missed menstrual cycle or positive home pregnancy test. This adjustment can be accomplished by increasing LT4 by additional 2 tablets of LT4 per week. Further adjustments should be individualized as they are dependent on the etiology of maternal hypothyroidism, as well as the preconception level of TSH. Serum thyroid function tests should be monitored closely.
6. Hypothyroid patients (receiving LT4) who are planning pregnancy should have their dose adjusted by their provider in order to optimize serum TSH values to <2.5 mIU/L preconception.

Some controversial problems pointed out in this review have been addressed by the guidelines in the following manner.

1. Euthyroid women (not receiving LT4) who are TPOAb positive require monitoring for hypothyroidism during pregnancy.
2. Serum TSH should be evaluated every 4 weeks during the first half of pregnancy and at least once between 26 and 32 weeks gestation.
3. Isolated hypothyroxinemia should not be treated in pregnancy, because of the lack of a documented effect of this intervention.

Some controversial problems included in this review that remain unsolved or not addressed.

1. There is insufficient evidence to recommend for or against screening for TPO Ab in the first trimester of pregnancy, or treating TPO Ab positive euthyroid women with LT4.
2. There is insufficient evidence to recommend for or against universal TSH screening at the first trimester visit.

## Figures and Tables

**Table 1:** Intervention studies describing the efficacy of LT4 treatment during pregnancy.

Study	Design	Material	Intervention	Target TSH	Pregnancy complications	Offspring complications
Abalovich et al. [38]	Retrospective study of pregnant women with OH TSH > 5 mIU/L, T4 < 4.5 ug/dL and SH TSH > 5 mIU/L, T4 normal	114 women OH *n* = 52 SH *n* = 62 99 pregnancies conceived under euthyroidism, 51 under OH or SH	Treatment with LT4 before or during pregnancy as soon as OH or SH was diagnosed	Optimal treatment TSH < 4 mIU/mL Inadequate treatment TSH > 4 mIU/L	Among pregnancies conceived under OH or SH miscarriage rate inadequate versus adequate treated 60% versus 0% in OH and 71.4% versus 0% in SH	Among pregnancies conceived under OH or SH preterm deliveries rate inadequate versus adequate treated 20% versus 0% in OH and 7.2% versus 9.5% in SH

Hallengren et al. [71]	Prospective observational	63 pregnant women with OH treated with LT4	Adjustment of the LT4 dose	<2 mIU/L	Fetal loss 6% (2/32) of optimally treated patients versus 29% (9/31) of women treated inadequately	Not examined

Negro et al. [72]	Prospective randomized trial	984 euthyroid women with TSH levels <4.2 mIU/L	group A, *n* = 57 TPO Ab (+) women treated with LT4 initiated at median 10 weeks of gestation versus group B, *n* = 58 no treatment for TPO Ab (+) women versus groups C *n* = 869 control TPO Ab (−)	Dose of LT4 stable during pregnancy 0.5 ug/kg/d for TSH < 1.0 mIU/L 0.75 ug/kg/d TSH 1.0-2.0 mIU/L, 1 ug/kg/d TSH > 2.0 mIU/L or anti-TPO > 1500 kIU/L	The rate of miscarriage lower in intervention group A compared to group B (3.5% versus 13.8%, *P* < 0.05) and similar to controls (3.5% versus 2.4%, *P* = *ns*)	Not examined

CATS study Lazarus [73]	Prospective randomized trial	22,000 women within the 16th week of gestation	In the intervention group LT4 was initiated during pregnancy in women with FT4 values lower than the 2.5th percentile and/or TSH values above the 97.5th percentile. The control group received no intervention.	<2.5 mIU/L I trimester <3 mIU/L II and III trimester	No data	The primary outcome was the mean WPPS-III score and the percentage of offspring with IQ < 85 points. The primary intention to treat analysis revealed no significant differences. A secondary analysis excluded women noncompliant with LT4 and also revealed no difference in relative risk of full scale IQ being below 85 in the screen group compared to the control group.
